# Transcriptional and Post-Transcriptional Regulations of Amyloid-β Precursor Protein *(*APP*)* mRNA

**DOI:** 10.3389/fragi.2021.721579

**Published:** 2021-08-11

**Authors:** Kaoru Sato, Ken-ichi Takayama, Makoto Hashimoto, Satoshi Inoue

**Affiliations:** ^1^ Department of Systems Aging Science and Medicine, Tokyo Metropolitan Institute of Gerontology, Tokyo, Japan; ^2^ Department of Basic Technology, Tokyo Metropolitan Institute of Medical Science, Tokyo, Japan

**Keywords:** amyloid precursor protein, Alzheimer’s disease, dementia, transcription, post-transcription, RNA-binding protein, alternative splicing, microRNA

## Abstract

Alzheimer’s disease (AD) is an age-associated neurodegenerative disorder characterized by progressive impairment of memory, thinking, behavior, and dementia. Based on ample evidence showing neurotoxicity of amyloid-β (Aβ) aggregates in AD, proteolytically derived from amyloid precursor protein (APP), it has been assumed that misfolding of Aβ plays a crucial role in the AD pathogenesis. Additionally, extra copies of the *APP* gene caused by chromosomal duplication in patients with Down syndrome can promote AD pathogenesis, indicating the pathological involvement of the *APP* gene dose in AD. Furthermore, increased *APP* expression due to locus duplication and promoter mutation of *APP* has been found in familial AD. Given this background, we aimed to summarize the mechanism underlying the upregulation of *APP* expression levels from a cutting-edge perspective. We first reviewed the literature relevant to this issue, specifically focusing on the transcriptional regulation of *APP* by transcription factors that bind to the promoter/enhancer regions. *APP* expression is also regulated by growth factors, cytokines, and hormone, such as androgen. We further evaluated the possible involvement of post-transcriptional regulators of *APP* in AD pathogenesis, such as RNA splicing factors. Indeed, alternative splicing isoforms of *APP* are proposed to be involved in the increased production of Aβ. Moreover, non-coding RNAs, including microRNAs, post-transcriptionally regulate the *APP* expression. Collectively, elucidation of the novel mechanisms underlying the upregulation of *APP* would lead to the development of clinical diagnosis and treatment of AD.

## Introduction

Alzheimer’s disease (AD) is the most common form of age-related dementia and a complex neurodegenerative disorder, phenotypically featured with progressive impairment of memory, thinking, and behavior as along with cognitive decline (Bateman et al., 2012; Mucke and Selkoe, 2012; Kirova et al., 2015; Hashimoto et al., 2018). AD is pathologically characterized by the deposition of senile plaques and neurofibrillary tangles (NTFs) in the brain (DeTure and Dickson, 2019). Senile plaques are mainly composed of soluble amyloid-β (Aβ) peptides (Panza et al., 2019), which form aberrant aggregates exhibiting neurotoxicity in the brain; therefore, the crucial role in the AD pathogenesis is assumed to be played by misfolded Aβ, namely amyloid hypothesis. Notably, NTFs is mainly comprised of a hyperphosphorylated TAU protein, which has been implicated in major neurodegenerative diseases including AD, termed Tauopathy (Panza et al., 2019). Aβ is produced through sequential proteolytic processing of a transmembrane protein, Aβ precursor protein (APP) by the β-site APP-cleaving enzyme 1 (BACE1) and γ-secretase (Panza et al., 2019), through the amyloidogenic pathway. In addition to the amyloidogenic pathway, the majority of APP undergoes non-pathogenic processing mediated by sequential cleavage of α-secretase and γ-secretase (Nguyen, 2019). In this pathway, an N-terminal secreted form of APP (sAPPα) is generated that plays numerous roles in normal physiological functions in the brain, such as neuronal proliferation, differentiation, migration, and synaptic function (Nguyen, 2019; Dar and Glazner, 2020).

To decipher the pathogenesis of AD, several studies have addressed the populations in which genetic variations are known to cause AD. In humans, the *APP* gene is located on chromosome 21 with 18 exons and is alternatively spliced into multiple isoforms, of which three isoforms, APP695, APP751, and APP770 are primarily generated. APP695 is predominantly expressed in neurons, whereas the remainders are expressed rather ubiquitously (Dai et al., 2018). Almost all adults with Down syndrome (DS) display neuropathological changes of AD over 40 years of age due to extra copies of *APP* attributed to the trisomy of chromosome 21 (Wiseman et al., 2015; Antonarakis, 2017; Lott and Head, 2019). Furthermore, a genetic variation observed in individuals with small internal duplications within chromosome 21 can result in three *APP* copies in a rare familial trait known as duplication of *APP* and can lead to an early-onset AD (Rovelet-Lecrux et al., 2006; Sleegers et al., 2006; Kasuga et al., 2009; Thonberg et al., 2011; Hooli et al., 2012; Swaminathan et al., 2012). In contrast, partial trisomy of chromosome 21 lacking an extra copy of the *APP* gene does not promote AD (Prasher et al., 1998; Korbel et al., 2009). The *APP* copy number is also mosaically amplified in the neurons of late-onset sporadic AD brains (Bushman et al., 2015). Additionally, genomic variations within *APP* promoter can upregulate its expression thereby increasing the risk of AD (Prasher et al., 1998; Guyant-Maréchal et al., 2007). This implies that the genetic variations involving an increase in *APP* mRNA levels are associated with AD pathophysiology. Moreover, increased *APP* expression levels have been detected in the brain, particularly in the entorhinal cortex neurons containing neurofibrillary tangles in AD patients (Cohen et al., 1988; Higgins et al., 1988; Guttula et al., 2012). Together, *APP* expression levels can impact the pathological processes in AD. Here, we summarized the literature relevant to this issue, specifically focusing on both transcriptional and post-transcriptional regulation of *APP* mRNA, and examined their roles in AD pathogenesis.

## Transcriptional Regulation of *APP*

In this section, we summarized the annotated genomic features of the human *APP* promoter/enhancer and its transcriptional regulators.

### *APP* Promoter/Enhancer Activity

**FIGURE 1:**
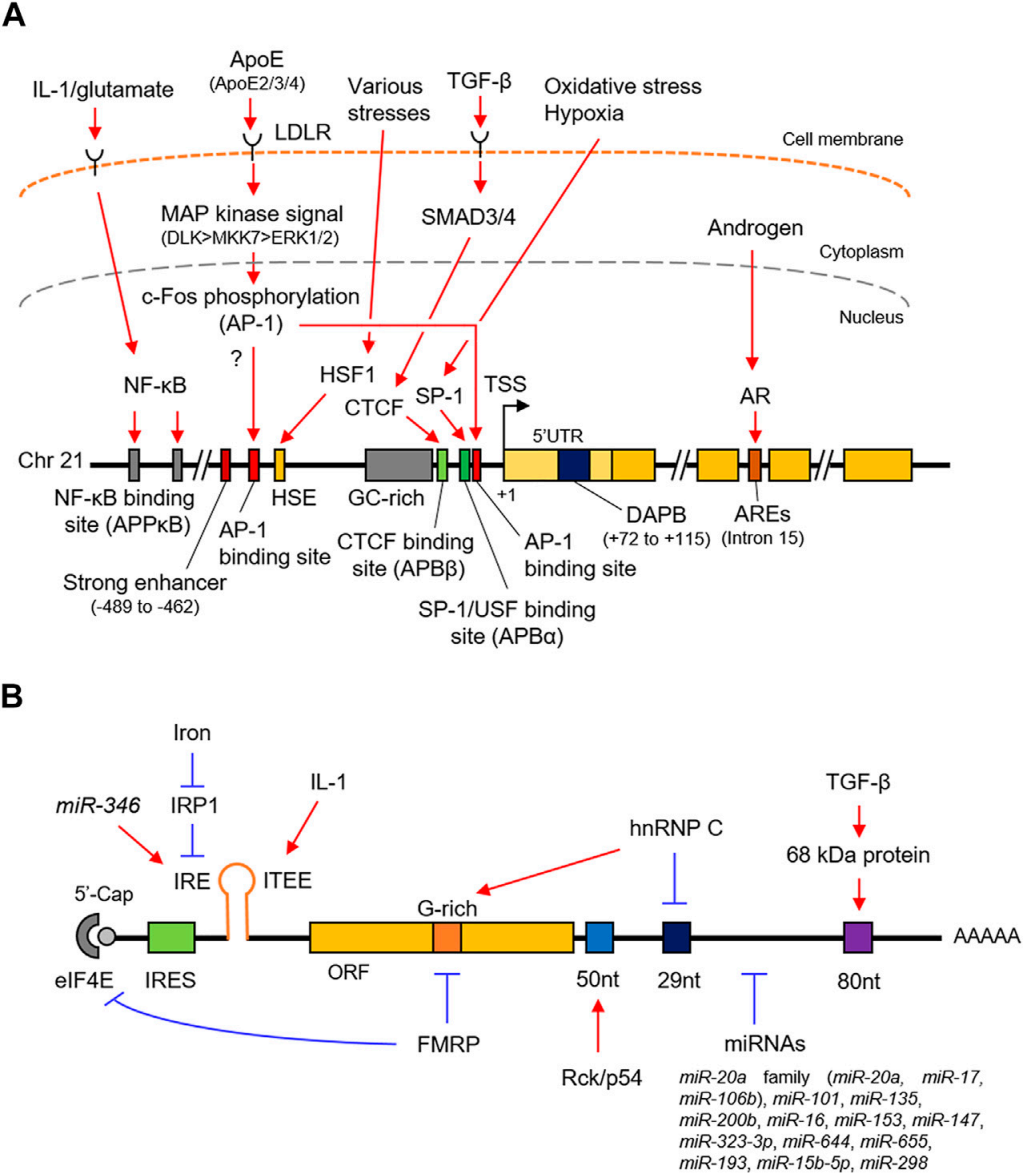
Schematic representation of the transcriptional and post-transcriptional regulation of *APP*. **(A)**. The promoter structure and regulating factors for the *APP* transcription. **(B)**. *cis-*regulatory elements and *trans-*acting factors for the post-transcriptional regulation. *APP*, amyloid-β precursor protein; SP-1, putative SP-1 binding site; AP-1, putative AP-1 binding site; HSP, heat shock element; DAPB, DNase I protected domain; ARE, androgen response element; AR, androgen receptor. IRES, internal ribosome entry site; IRE, iron responsive element; ITEE, interleukin-1 translation enhancer element; IRP1, iron response protein 1; 52 nt, 52 nt element; 29 nt, 29 nt element; 80 nt, 80 nt element.

The promoter of the human *APP* lacks TATA and CAAT boxes upstream of the transcription start site but contains a high GC region with five GGGCGC boxes (Lahiri and Robakis, 1991) (Figure 1A), which adapts to the typical characteristics of a housekeeping gene (Smale and Kadonaga, 2003). The proximal region of the promoter from –150 to –10 base pairs (bp) contains the minimum essential elements for *APP* promoter activity (Lahiri and Robakis, 1991; Lahiri and Nall, 1995). The region from − 600 to − 460 bp acts as a transcriptionally positive regulator; in particular, a 26 bp positioned between –489 and –462 bp acts as a strong enhancer. In contrast, the region from –450 to –150 bp works as a negative regulator. Additionally, the downstream region of the *APP* promoter does not match the consensus sequences for any of the downstream core promoter sequences, such as the downstream promoter element (DPE), which is generally required for efficient transcription (Vostrov et al., 2010). However, the region from + 72 to + 115 has an unknown nuclear factor-binding domain termed as DNase I protected domain (DAPB), which is required for *APP* promoter activity in HeLa cells.

### Stimulating Protein 1 (SP-1)

SP-1 belongs to the Sp/KLF family of transcription factors and directly binds to the DNA sequence by its own zinc finger motif to enhance gene transcription in response to oxidative stress and hypoxia (Yeh et al., 2011). Indeed, SP-1 binds to the *APP* promoter (Figure 1A and Table 1) and accelerates the production of *APP* transcripts (Pollwein, 1993; Hattori et al., 1997; Basha et al., 2005). The SP-1 binding site partially overlaps with the *APP* promoter binding α (APBα) domain (Figure 1A), an intact nuclear factor-binding site essential for *APP* transcription. Additionally, the upstream transcription factor (USF), a basic helix-loop-helix transcription factor, also binds to the APBα domain Vostrov et al. (1995) and contributes to an increased *APP* expression in neurons (Yang et al., 1999).

**TABLE 1:** List of transcriptional and post-transcriptional regulators for the *APP* expression.

Name	Mechanism	*APP* level	References
Transcription			
SP-1	Activate *APP* transcription through binding to the SP-1 element within the *APP* promoter	Up	Lahiri and Robakis, (1991)
			Pollwein, (1993)
			Lahiri and Nall, (1995)
			Hattori et al. (1997)
AP-1	Activate *APP* transcription through binding to the AP-1 element within the *APP* promoter	Up	Trejo et al. (1994)
			Lahiri and Ge, (2004)
CTCF	Activate *APP* transcription through binding to the APBβ domain within the *APP* promoter	Up	Quitschke and Goldgaber, (1992)
			Quitschke, (1994)
			Quitschke et al. (2000)
HSF1	In response to stress, activate *APP* transcription through binding to the HSE within the *APP* promoter	Up	Dewji et al. (1995)
			Dewji and Do, (1996)
NF-κB/Rel	Activate *APP* transcription through binding to two binding motifs at the distal *APP* promoter	Up	Grilli et al. (1995)
			Grilli et al. (1996)
USF	Activate *APP* transcription through binding to the APBα domain within the *APP* promoter	Up	Vostrov et al. (1995)
			Yang et al. (1999)
Androgen	Activate *APP* transcription through binding to ARE within the *APP* intron	Up	Takayama et al. (2009)
			Takayama et al. (2019)
ApoE	Activate MAP kinase signal and c-Fos phosphorylation (AP-1)	Up	Huang et al. (2017)
Post-transcription			
Iron	Dissociate IRP1 from IRE by binding, eliciting the *APP* translation	Down	Rogers et al. (2008)
			Cho et al. (2010)
IL-1	Bind to the IL-1 translational enhancer element at the *APP* 5′UTR, increasing its stability	Up	Rogers et al. (1999)
TGF-β	Modulate the 81 nt element-mediated stabilization of *APP* mRNA	Up	Amara et al. (1999)
FMRP	Bind to the G-rich motif, inhibiting the *APP* translation	Down	Westmark and Malter, (2007)
	Inhibit eIF4E with CYFIP1		Napoli et al. (2008)
hnRNP C	Bind to the G-rich motif and the 29 nt element, increasing the *APP* mRNA stability	Up	Rajagopalan et al. (1998)
Rck/p54	Bind to the 52 nt element, increasing the *APP* mRNA stability	Up	Broytman et al. (2009)
PSF/SFPQ	Stabilize *APP* mRNA through interaction	Up	Takayama et al. (2019)
miR-106a (*)	Bind to 3′UTR of *APP* transcript, and lead the miRNA-mediated repression	Down	Patel et al. (2008)
miR-520c (*)			Hébert et al. (2009)
miR-20a family (miR-20a, miR-17, miR-106b) (*)			Vilardo et al. (2010)
miR-101 (*)			Delay et al. (2011)
miR-135 (*)			Long and Lahiri, (2011)
miR-200b (*)			Liang et al. (2012)
miR-193 (*)			Long et al. (2012)
miR-298 (*)			Barbato et al. (2014)
miR-16			Liu et al. (2014a)
miR-153			Liu et al. (2014b)
miR-147			Galimberti et al. (2014)
miR-323-3p			Zhang et al. (2015)
miR-644			Liu et al. (2019)
miR-655			Chopra et al. (2020)
miR-15b-5p			
miR-346 (*)	Bind to IRE in the *APP* 5′UTR and upregulate its translation	Up	Long et al. (2019)

Asterisk (*) indicates miRNA of which functional involvement has been investigated using human brain tissue or neuronal cells.

### Activator Protein-1 (AP-1)

AP-1 is a heterodimer composed of proteins belonging to the c-Fos, c-Jun, activating transcription factors (ATFs), and Jun dimerization protein (JDP) families (Shaulian and Karin, 2002). AP-1 regulates gene expression in response to numerous stimuli, including cytokines, growth factors, and stress (Shaulian and Karin, 2002; Hess et al., 2004). Although two putative AP-1 recognition sites are located at the *APP* promoter, the distal AP-1 recognition site alone is sufficient for transcriptional activation by AP-1, such as c-Fos/-Jun heterodimer, rather than the proximal site (Trejo et al., 1994; Lahiri and Ge, 2004) (Figure 1A and Table 1).

### CCCTC-Binding Factor (CTCF) and Transforming Growth Factor-β (TGF-β)

CTCF is generally a multifunctional positive or negative regulator of various target genes and plays a key role in transcriptional insulation (Quitschke et al., 2000). CTCF contains tandem 11 zinc finger motifs, of which five to seven zinc fingers are required for binding to positions –98 and –83 bp of the human *APP* promoter, which was formerly designated as APBβ, another intact nuclear factor-binding site essential for *APP* transcription (Quitschke and Goldgaber, 1992; Quitschke, 1994; Quitschke et al., 2000) (Figure 1A and Table 1). APBβ is also responsive to TGF-β, a multifunctional cytokine, in which two TGF-β signaling mediators, mothers against decapentaplegic homolog 3 (SMAD3) and SMAD4, associate with CTCF on the APBβ and promote *APP* transcription in cooperation with SP-1 (Burton et al., 2002; Docagne et al., 2004).

TGF-β is also implicated in the post-transcriptional regulation of *APP*. A TGF-β-responsive protein forms a 68 kDa RNA-protein complex and is proposed to stabilize the *APP* transcript by binding to the 81 nt sequence within the *APP* 3′UTR, increasing its translation (Amara et al., 1999; Westmark and Malter, 2012).

### Heat Shock Transcription Factor 1 (HSF-1)

The transcription of *APP* is stimulated by various stress factors such as heat shock and treatment with ethanol and arsenite (Dewji et al., 1995). The HSF1 binds to the heat shock element (HSE) of the *APP* promoter (Dewji and Do, 1996) (Figure 1A and Table 1). It acts as a primary mediator of stress-responsive transcription of pro-survival genes, including heat shock proteins (Akerfelt et al., 2010). Under normal conditions, HSF1 is predominantly localized in the cytoplasm in a repressed monomeric form. Upon stress, HSF1 trimerizes and accumulates in the nucleus, where it binds to HSE.

### Nuclear Factor (NF)-κB/Rel

The NF-κB transcription factor forms a dimer composed of NF-κB/Rel family subunits including NF-κB1/p50 and regulates transcription of genes involved in immune and inflammatory responses in response to stimuli such as inflammation and disease (Dresselhaus and Meffert, 2019). NF-κB1/p50-containing complex, widely expressed in neurons and glial cells in the human brain, is activated through the canonical NF-κB pathway extracellularly stimulated by cytokines and neurotransmitter in neurons, where it plays important roles for neuroprotective functions including anti-apoptosis under the neurodegenerative condition, but not well-defined in glial cells. To activate *APP* transcription, the NF-κB1/p50-containing complex specifically recognize APPκB sites in the distal *APP* promoter, where IL-1 and glutamate enhance its binding activity (Grilli et al., 1995; Grilli et al., 1996) (Figure 1A and Table 1).

### Androgen

Androgens are natural steroid hormones that regulate various physiological phenomena taking place in several tissues and organs, including the brain, by binding to the androgen receptor (AR), a member of the nuclear receptor superfamily (Chang et al., 2013; Takayama and Inoue, 2013). After ligand binding, the AR homodimer translocates into the nucleus, where it binds to the androgen-responsive element (ARE), and subsequently activates gene expression often with epigenetic changes in the chromatin state (Tewari et al., 2012; Nevedomskaya et al., 2016; Nadal et al., 2017; Stelloo et al., 2019). *APP* is a primary androgen-regulated gene in human neuronal and prostate cancer cells (Takayama et al., 2009; Takayama et al., 2019). In neurons, AR directly binds to the ARE located within the genomic regions corresponding to the 15th intron of the *APP* gene (Takayama et al., 2019) (Figure 1A and Table 1). Notably, the chromatin binding level of histone H3 acetylated at lysine 9 (H3K9ac), a transcriptionally active histone mark, at the *APP* promoter is enhanced with overexpression of AR. Importantly, the androgen concentration declines with age in serum and brain (Rosario et al., 2011; Grimm et al., 2016; Gaignard et al., 2017) and is likely associated with AD development (Gouras et al., 2000; Wahjoepramono et al., 2008; César et al., 2016; Jayadevappa et al., 2019).

### Apolipoprotein E (ApoE)

ApoE is a major component of low-density lipoprotein (LDL) and very-low-density lipoprotein (VLDL). It acts on the metabolism of fats, including the transportation of lipids, fat-soluble vitamins, and cholesterol into the lymph and blood by binding to the LDL receptor (LDLR) (Goldstein and Brown, 2015). Although it is largely synthesized in the liver, the elevated expression has also been characterized in the brain, primarily in the astrocytes (Holtzman et al., 2012; Wang and Eckel, 2014). Humans harbor three major ApoE alleles: ApoE-ε2, ApoE-ε3, and ApoE-ε4 (Ghebranious et al., 2005; Eisenberg et al., 2010). Importantly, ApoE-ε4 is a well-known genetic risk factor for atherosclerosis and AD in the brain, unlike ApoE-ε2 and ApoE-ε3 (Strittmatter et al., 1993; Farrer et al., 1997; Huang et al., 2017). In the human brain, ApoE proteins secreted from the glia stimulate *APP* transcription and Aβ production with different efficacy in the neurons (Huang et al., 2017; Vogrinc et al., 2021). In this pathway, secreted ApoE protein binds to the LDLR on the neuron surfaces and activates a non-canonical MAP kinase signaling pathway mediated by DLK, MKK7, and ERK1/2 (Figure 1A and Table 1). Subsequently, c-Fos, a subunit of AP-1, is phosphorylated (Shaulian and Karin, 2002), in turn enhances AP-1–dependent *APP* transcription (Huang et al., 2017). Notably, ApoE-ε4 is also involved in the recognition and engulfing of Aβ in the brain (Strittmatter et al., 1993; Farrer et al., 1997).

## Post-Transcriptional Regulation of *APP*

We have reviewed the *cis-*regulatory elements of the *APP* transcript and *trans*-acting factors for the post-transcriptional regulation of *APP*.

### Iron

The *APP* 5′untranslated region (UTR) contains an internal ribosome entry site (IRES), an iron-responsive element (IRE), and an interleukin-1 (IL-1) translation enhancer element (Figure 1B and Table 1). The IRES is a specialized RNA element that allows the recruitment of eukaryotic ribosomes to mRNA, regardless of the presence of the 5′cap (Hellen and Sarnow, 2001; López-Lastra et al., 2005); therefore, endogenous *APP* is translated in a cap-independent manner (Beaudoin et al., 2008). In the absence of iron, iron response protein 1 (IRP1), an iron-dependent translational repressor, is presumed to bind to the IRE of the *APP* transcript. This prevents the recruitment of the 40 S ribosome at the 5’cap, subsequently repressing the translation (Rogers et al., 2008; Cho et al., 2010).

### Interleukin-1 (IL-1)

An IL-1 translation enhancer element (ITEE), also known as IL-1 acute box, is present close to IRE, (Rogers et al., 2002; Rogers et al., 2008; Ruberti et al., 2010; Bandyopadhyay et al., 2013) (Figure 1B and Table 1). IL-1α and IL-1β, are a group of IL-1 cytokines that play a crucial role in regulating immune and inflammatory responses to not only infections but also in all inflammatory, physiological or pathological phenomena (Dinarello, 2018; Kaneko et al., 2019). It is also known to be increased in the brains of AD patients (Cacabelos et al., 1994; Shaftel et al., 2008; Italiani et al., 2018). Using CAT assay, a 90 nt element in the *APP* 5′UTR, which includes the enhancer element, was found to enhance its translation without changing the steady-state mRNA level (Rogers et al., 1999; Westmark and Malter, 2012), indicating that IL-1 post-transcriptionally upregulates *APP* translation.

### Fragile X Mental Retardation Protein (FMRP)

The protein-coding region of *APP* contains a G-rich motif that interacts with the FMRP (Figure 1B and Table 1) which is highly expressed in the brain. Loss of FMRP causes fragile X syndrome, largely characterized by cognitive impairment (O'Donnell and Warren, 2002). FMRP associates with the *APP* transcript *via* its own multiple RNA-binding motifs such as KH motif and RGG box to repress the translation of *APP* in a type 1 metabotropic glutamate receptor (mGluR)-dependent manner; thus, stimulation of mGluR elicits an increase in the *APP* translation (Westmark and Malter, 2007). In addition, FMRP interacts with cytoplasmic FMR1–interacting protein 1 (CYFIP1), thereby inhibiting *APP* translation by sequestering eukaryotic initiation factor 4E (eIF4E) (Napoli et al., 2008). Notably, loss of FMRP results in the production of excess soluble APP, which contributes to a deficiency in dendrite maturation (Pasciuto et al., 2015). Normalizing *APP* levels in Fmrp-knockout mice can rescue the fragile X phenotypes (Westmark et al., 2011), indicating the importance of *APP* homeostasis in the development of this disorder.

### Heterogeneous Nuclear Ribonucleoprotein C (hnRNP C)

It has been reported that hnRNP C, a ubiquitous RNA regulatory protein, competitively binds to the same G-rich motif as FMRP (Figure 1B and Table 1). It harbors an RNA recognition motif (RRM) and is associated with pre-mRNAs to regulate RNA processing, metabolism, and transport (Piñol-Roma and Dreyfuss, 1993; Han et al., 2010). In contrast to FMRP, hnRNP C enhances *APP* translation by binding to the G-rich motif. Additionally, hnRNP C also binds to the repressive 29 nt element in the *APP* 3′UTR, thereby increasing its stability (Rajagopalan et al., 1998).

### Rck/p54

Rck/p54, a member of the DEAD-box family of RNA helicases is also known as DEAD-box helicase 6 (DDX6). Rck/p54 modulates mRNA secondary structures Akao et al. (1995) by binding to the 52 nt elements downstream of the stop codon and increasing *APP* mRNA stability (Broytman et al., 2009) (Figure 1B and Table 1). Indeed, the helicase activity of Rck/p54 is required for *APP* mRNA stability.

### Polypyrimidine Tract-Binding Protein-Associated Splicing Factor/Splicing Factor Proline- and Glutamine-Rich (PSF/SFPQ)

PSF, also known as SFPQ, is a ubiquitously expressed nuclear RNA-binding protein (RBP) (Knott et al., 2016). PSF/SFPQ is mainly localized at the nucleus, in particular to the membraneless condensates known as paraspeckles, in which nuclear enriched abundant transcript 1 (NEAT1), an architectural long non-coding RNA, is bound to core proteins, including PSF/SFPQ. The latter regulates various cellular mechanisms such as alternative splicing and nuclear retention of mRNAs (Nakagawa et al., 2018; Lim et al., 2020). Importantly, PSF/SFPQ plays a critical role in neural development as well as in neurodegenerative diseases, including AD (Ke et al., 2012; Lu et al., 2018; Younas et al., 2020). It directly binds to the primary *APP* transcripts in human neuronal cells, leading to *APP* mRNA stabilization (Takayama et al., 2019) (Figure 1B and Table 1).

### microRNA (miRNA)

RNA silencing is a nucleotide-sequence-specific regulation of gene expression mediated by small non-coding RNAs such as miRNAs (Ghildiyal and Zamore, 2009; Kim et al., 2009; Czech and Hannon, 2011). In most cases, miRNAs interact with the 3′UTR of target mRNAs to induce their degradation by mRNA decay or inhibit their translation. In recent years, several miRNAs have been identified to participate in AD pathogenesis by regulating the expression of multiple target genes, including *APP*. miR-106a and miR-520c were the first miRNAs experimentally demonstrated to downregulate *APP* levels post-transcriptionally (Patel et al., 2008). In subsequent studies, further 15 miRNAs, as listed in Table 1, have been identified to bind directly to the 3′UTR of the human *APP* transcript and downregulate its expression at the post-transcriptional level. Unlike most miRNAs, miR-346 interacts with the *APP* 5′UTR to promote translation (Long et al., 2019) (Figure 1B). The miR-346 target site overlaps with the IRE at the *APP* 5′UTR, where miR-346 would displace IRP1 even at low iron levels, eliciting *APP* translation, suggesting that miR-346 may maintain *APP* homeostasis and prevent the pathogenic *APP* cascade in AD.

## Conclusion

Here, we summarized multiple regulatory mechanisms of *APP* mRNA at the transcriptional and post-transcriptional levels, particularly in the human brain. An imbalance in *APP* levels caused by aberrancy of these mechanisms can trigger increased AD development, as mentioned above.

## Perspective

Immunotherapy against ApoE improves amyloid-associated phenotypes rather than Aβ (Xiong et al., 2021), suggesting elucidation of the regulatory mechanisms of *APP* expression can provide effective therapeutic strategies to interrupt the development or further progression of AD. Moreover, recent advances in high-throughput sequencing technologies have facilitated the reconstruction of the entire transcriptional landscape and RNA–RBP networks in human diseases. In addition, more recently, RBPs such as transactive response DNA-binding protein 43 (TDP-43) and fused in sarcoma (FUS) related to neurodegenerative diseases have been shown to undergo liquid-liquid phase separation (LLPS). Aberrant phase transitions of these RBPs in the brain lead to the disorder (Patel et al., 2015; Wolozin and Ivanov, 2019; Boyko et al., 2020), suggesting a possible role of LLPS in AD-related RBPs in *APP* homeostasis.
